# HSV-1 selectively packs the transcription factor Oct-1 into EVs to facilitate its infection

**DOI:** 10.3389/fmicb.2023.1205906

**Published:** 2023-06-15

**Authors:** Yilei Ma, Xiaomei Deng, Lingyue Zhou, Hongchang Dong, Pei Xu

**Affiliations:** The Centre for Infection and Immunity Studies, School of Medicine, Sun Yat-sen University, Shenzhen, Guangdong, China

**Keywords:** Oct-1, POU2F1, HSV-1, EVs, extracellular vesicle, subcellular localization

## Abstract

HSV-1 hijacks the cellular vesicular secretion system and promotes the secretion of extracellular vesicles (EVs) from infected cells. This is believed to facilitate the maturation, secretion, intracellular transportation and immune evasion of the virus. Intriguingly, previous studies have shown that noninfectious EVs from HSV-1-infected cells exert antiviral effects on HSV-1 and have identified host restrictive factors, such as STING, CD63, and Sp100 packed in these lipid bilayer-enclosed vesicles. Octamer-binding transcription factor-1 (Oct-1) is shown here to be a pro-viral cargo in non-virion-containing EVs during HSV-1 infection and serves to facilitate virus dissemination. Specifically, during HSV-1 infection, the nuclear localized transcription factor Oct-1 displayed punctate cytosolic staining that frequently colocalized with VP16 and was increasingly secreted into the extracellular space. HSV-1 grown in cells bereft of Oct-1 (Oct-1 KO) was significantly less efficient at transcribing viral genes during the next round of infection. In fact, HSV-1 promoted increased exportation of Oct-1 in non-virion-containing EVs, but not the other VP16-induced complex (VIC) component HCF-1, and EV-associated Oct-1 was promptly imported into the nucleus of recipient cells to facilitate the next round of HSV-1 infection. Interestingly, we also found that EVs from HSV-1-infected cells primed cells for infection by another RNA virus, vesicular stomatitis virus. In summary, this investigation reports one of the first pro-viral host proteins packed into EVs during HSV-1 infection and underlines the heterogenetic nature and complexity of these noninfectious double-lipid particles.

## Introduction

Human herpes simplex virus type 1 (HSV-1) is a double-stranded DNA virus that causes cold sores in more than 67% of the population worldwide (James et al., 2020). Once contracted, the virus lytically replicates in the epithelial cells and remains dormant in the peripheral neurons for a lifetime with sporadic reactivation. During HSV-1 lytic replication, viral genes are sequentially transcribed from the viral genome and are thus classified into immediate early (α), early (β), and late (γ) genes based on their strict chronological order of expression. The VP16-induced complex (VIC), consisting of host cell factor 1 (HCF-1), octamer transcription factor-1 (Oct-1/POU2F1) and viral tegument protein VP16, is essential in initiating HSV-1 lytic replication by derepressing the transcription of immediate early genes. Upon HSV-1 entry, HCF-1 interacts with VP16 through the 6 Kelch domains on its N-terminus (amino acid sequence 3-455 aa) and mediates the nuclear importation of VP16 (Wysocka and Herr, 2003). In the nucleus, the host protein Oct-1 binds to the HCF-1 and VP16 complex through its POU-specific domain (POU_S_) and recognizes the VP16-responsive sequence (TAATGARAT) in the promoter of five HSV-1 α genes (ICP0, 4, 22, 27, 47), activating their transcription by recruiting lysine-specific demethylase 1 (LSD1) to demethylate histones bound to the α promoters (Liang et al., 2009; Zhou et al., 2010; Roizman and Whitley, 2013; Ding et al., 2022). Transcription of α genes then activates the remainder of the viral genes, launching the lytic replication cycle of the virus.

Oct-1, one of the key components of VIC, continues to participate in HSV-1 infection beyond this initial stage. The protein is an ubiquitously expressed member of the POU factors and recognizes the consensus “octamer motif” [ATGC (A/T)AAT] and variants thereof (Ingraham et al., 1988; Sturm et al., 1988; Kemler et al., 1989; Pance, 2016; Vázquez-Arreguín and Tantin, 2016). It possesses broad functions in tumor initiation/progression (Vázquez-Arreguín and Tantin, 2016; Verhasselt et al., 2022), immune modulation (Shakya et al., 2015; Kim et al., 2019), cellular proliferation (Magné et al., 2003; Kang et al., 2009), stress response (Fan et al., 2002; Tantin et al., 2005; Kang et al., 2009), metabolic regulation (Shakya et al., 2009) and stem cell function (Tantin, 2013) through direct DNA binding and/or various interacting partners. However, the role of Oct-1 at later stages of HSV-1 infection may be less related to its DNA binding capability. Studies utilizing Oct-1-deficient mouse embryonic fibroblast (MEF) cells also reveal that the protein is required for efficient HSV-1 replication factory assembly, independent of its function in HSV-1 α gene transcription (Nogueira et al., 2004). Furthermore, Oct-1 is significantly post-translationally modified by HSV-1 late in infection to reduce its affinity to the “octamer motif,” and this is believed to contribute to α gene shutoff by the virus (Kemler et al., 1991; Advani et al., 2003). Oct-1 is also closely linked to both HSV-1 latency establishment and reactivation. Prior studies show that Oct-1 is targeted by neuronal miR-138 to favor latency (Sun et al., 2021) and that Oct-1 participates in promoting viral mRNA transcription during HSV-1 reactivation in mice sensory neurons (Kim et al., 2012).

Extracellular vesicles (EVs) are a heterogeneous group of vesicles that are released from cells and serve as a means of transferring biological materials between cells (Pan and Johnstone, 1983; Harding et al., 1984; Wubbolts et al., 2003; Skog et al., 2008; Trajkovic et al., 2008; Robinson et al., 2011; Bellingham et al., 2012; Llorente et al., 2013; Raposo and Stoorvogel, 2013; Kalamvoki et al., 2014; Yáñez-Mó et al., 2015; Deschamps and Kalamvoki, 2018; Ma et al., 2022). These small, membrane-bound structures are actively involved in various physiological processes, including the immune response, development, cell-to-cell signaling and infection (Becker et al., 2016; Chen et al., 2018; van der Grein et al., 2018; Huang et al., 2019). EVs and their secretion pathways are deeply intertwined in the life cycle of herpesviruses. Members from the three subfamilies, alpha-, beta-and gamma-, modulate the extracellular vesicle secretion pathways to facilitate progeny virus exit from cells (Bello-Morales and López-Guerrero, 2018). Moreover, recent studies of the role of non-virion-containing EVs in HSV-1 infection further revealed their complex composition and cellular origins and suggested their delicately poised role in modulating virus dissemination (Kalamvoki and Deschamps, 2016). Similar to members of retroviruses (Mack et al., 2000; Sheehy et al., 2002; Newman et al., 2005) and hepatitis viruses (Feng et al., 2013; Ramakrishnaiah et al., 2013; Bukong et al., 2014; Chahar et al., 2015; Feng et al., 2015), HSV-1 has been reported to utilize EVs to package and deliver viral components or infectious virions to facilitate or achieve infection or to expand cell tropism (Bello-Morales et al., 2018; Bello-Morales and López-Guerrero, 2018). An endosomal sorting complex required for transport (ESCRT)-positive EV population containing viral factors such as VP16, Us11, and various viral glycoproteins has been isolated and shown to promote HSV-1 infection (Dogrammatzis et al., 2021). Conversely, EVs secreted by HSV-1-infected cells collectively have a negative effect on HSV-1 infection. A large and distinct population of virus-induced EVs that lack ESCRT but carry STING and CD63 has been shown to strongly inhibit virus spread, and both host factors have been individually demonstrated to exert antiviral roles through EV delivery (Kalamvoki et al., 2014; Deschamps and Kalamvoki, 2018; Dogrammatzis et al., 2019). Our group recently reported that during HSV-1 infection, there is an increase in the secretion of Sp100A into the extracellular space via EVs, which contributes to antiviral responses (Chen et al., 2022).

To date, no pro-viral host factors have been identified within extracellular vesicles derived from herpes simplex virus type 1 (HSV-1)-infected cells, which represents an intriguing phenomenon. Considering the critical role of the VIC complex and that only VP16 is a tegument protein packed in the virions, we investigated whether the virus promoted secretion of the other two host proteins to enhance infection. We initially observed that endogenous Oct-1 is increasingly secreted into the extracellular environment by HSV-1-infected cells and that HSV-1 infection led to increased Oct-1 protein levels in the culture medium but not HCF-1. Interestingly, during late lytic HSV-1 infection, colocalization of Oct-1 and VP16 was detected in the perinuclear area of infected cells. To determine the role of Oct-1 in natural HSV-1 infection, we subsequently utilized clustered regularly interspaced short palindromic repeats (CRISPR)/Cas9 technology to generate single-cell derived clones bereft of Oct-1 expression (Oct-1 KO cells) and made the following observations: (I) the deletion of Oct-1 significantly hindered HSV-1 infection; (II) the supernatant collected from infected HEp-2 cells facilitated viral gene transcription in recipient cells to a greater extent than that collected from infected Oct-1 KO cells; (III) both Oct-1 and HCF-1 were released into the extracellular space via extracellular vesicles (EVs), with the secretion of Oct-1 being increased during HSV-1 infection; (IV) Oct-1 could be transmitted into recipient cells via EVs; (V) Oct-1 from the EV donor cells facilitated HSV-1 replication in cells receiving EVs, regardless of whether the donor cells were infected or not; and (VI) EVs released by HSV-1-infected cells facilitated VSV replication, but this effect was independent of Oct-1.

## Materials and methods

### Cell lines and viruses

HEp-2 and Vero cells were maintained in Dulbecco’s modified Eagle’s medium (DMEM; Corning, 10-013-CVRC) supplemented with 10% fetal bovine serum (FBS; Gibco, cat #10270–106). The Oct-1 KO cell line was constructed from HEp-2 through the CRISPR technique by designing sgRNA sequences targeting different exon regions of Oct-1 and the corresponding homologous arm sequences. Mycoplasma contamination routinely tested negative for all cell lines. HSV-1(F), abbreviated as HSV-1 in the study, is the prototype strain used in the laboratory, which was amplified in HEp-2 cells and titrated in Vero cells by plaque assay.

### Antibodies and drugs

The antibodies and drugs used in this study included rabbit monoclonal anti-Oct-1 antibody (Abcam, ab178869), rabbit monoclonal anti-HCF-1 antibody (Abcam, ab289975), mouse monoclonal anti-VP16 antibody (Santa Cruz, sc7545), mouse monoclonal anti-β-actin antibody (Sino Biological, 1000166), rabbit monoclonal anti-GAPDH antibody (Abways Technology, AB0037), mouse monoclonal anti-Histone-H3 antibody (Sino Biological, 100005-MM01), rabbit polyclonal antibody anti-TSG101 (Proteintech, 28283-I-AP), mouse monoclonal anti-HSV-1-gD antibody (Santa Cruz, sc21719), mouse monoclonal anti-ICP0 antibody (Santa Cruz, 13,118), Alexa Fluor 594-labeled goat anti-rabbit IgG (H + L; Invitrogen, 2165334), Alexa Fluor plus 488-labeled goat anti-mouse IgG (H + L; Invitrogen, A32723), goat anti-mouse IgG-HRP (Invitrogen, 31430), goat anti-rabbit IgG (H + L)-HRP (Invitrogen, 32460), and protease inhibitor cocktail (Thermo Scientific, EO0492).

### Oct-1 KO cell line construction

HEp-2 cells were transfected with CRISPR plasmids along with sgRNA by JetPRIME at a ratio of 1:2. At 48 h post transfection, cells were screened by blasticidin (2 μg/mL, Gibco, A1113903) until the control group exhibited complete cell death. Then, the remaining cells were proliferated for monoclonal screening in 96-well plates. Oct-1 expression was evaluated in each single-cell clone by immunofluorescence staining and immunoblot analyses, and then the complete Oct-1 knockout clone was proliferated in DMEM supplemented with 10% FBS and blasticidin (2 μg/mL).

### EV purification by density gradient ultracentrifugation

The experiments were conducted as described previously (Deschamps and Kalamvoki, 2018). The FBS used for cell culture in this set of experiments was depleted of EVs through ultracentrifugation. Briefly, 2 × 10^7^ cells were infected with HSV-1 at a multiplicity of infection (MOI) of 0.1. At 50 hpi, the culture medium was collected and centrifuged at 300 g for 5 min and then at 2,000 g for 20 min prior to filtration through a 0.45 μm pore size filter. The flow through was then concentrated to 1,000 μL using an Amicon Ultra-15 mL, 30 kDa Centrifugal Filter Unit (Merck, UFC903024) and loaded on top of an iodixanol/sucrose gradient ranging from 6% to 18%, with a 1.2% increment. Samples were centrifuged in an SW40 Ti rotor for 16 h at 160,000 g and 4°C. Fractions in 500 μL were collected from top to bottom, numbered 1 to 24 and analyzed.

### Transmission electron microscopy

In brief, 10 μL of fixed extracellular vesicles were dropped on parafilm and incubated for 1 min on a discharged carbon-filmed grid. Grids were blotted up with filter paper as dry as possible and finally incubated for 1 min in 1% uranyl acetate and allowed to dry to be analyzed using a JEM-1400 flash electron microscope (JEOL Ltd., Japan).

### Subcellular fractionation

Subcellular fractionation was performed using NE-PER™ Nuclear and Cytoplasmic Extraction Reagents (Thermo Scientific, 78,835) according to the manufacturer’s protocol. In short, 1 × 10^6^ cells were collected, pelleted by centrifugation at 500 g for 5 min, resuspended in 100 μL of ice-cold CER I (supplemented with 10 μL/mL protease inhibitor) and incubated on ice for 10 min. Then, 5.5 μL of ice-cold CER II was added and incubated for 1 min on ice. The samples were centrifuged for 5 min at maximum speed in a microcentrifuge (~16,000 × g) to pellet nuclei, and supernatants were collected as cytosolic membrane-associated components. The pellets were washed and resuspended in 50 μL of ice-cold NER (supplemented with 10 μL/mL protease inhibitor) on ice for 40 min, vortexed for 15 s every 10 min, and sonicated (10 s, 20% amplification). The final solution contained the extract comprising nuclear membranes and nuclear proteins.

### Immunofluorescence staining and immunoblot analyses

For immunofluorescence, cells cultured on slides were washed with PBS, fixed and permeabilized in methanol at −80°C overnight. Cells were then blocked with PBS-TBH (10% FBS, 1% BSA, 1× PBS, 0.1% Triton™ X-100) at room temperature for 30 min, incubated with primary antibodies at appropriate dilutions in PBS-TBH overnight at 4°C and then with fluorophore-conjugated secondary antibodies at appropriate dilutions in PBS-TBH for 30 min at 37°C in the dark, with extensive washes in between the steps. The slides were mounted using mounting medium with DAPI (Abcam, 104139).

For immunoblotting, to obtain whole-cell extracts, we harvested cells in RIPA buffer and then sonicated the whole cell lysates two times on ice at 20% power and 10s each time to solubilize everything. And whole-cell extracts or subcellular fractions were subjected to SDS-PAGE separation, transferred onto PVDF membranes, blocked with 5% BSA in PBS with 1% Tween-20, incubated with primary and secondary antibodies and developed using SuperSignal West Pico PLUS Chemiluminescent substrate (Thermo Fisher, 34580).

### RNA extraction and qRT-PCR

Total RNA was extracted using an OMEGA RNA extraction kit (Omega, R6834-01) according to the manufacturer’s protocol. Gene expression levels were quantified by qRT-PCR (StepOnePlus™, Thermo Fisher) using the SYBR Green detection system (Accurate Biology, AG11701) and normalized to GAPDH. The primer information is as follows: ICP27-F “CGGGCCTGATCGAAATCCTA,” ICP27-R “GACACGACTCGAACACTCCT,” TK-F“CTTAACAGCGTCAACAGCGTGCCG,” TK-R “CCAAAGAGGTGCGGGAGTTT,” VP16-F “CCATTCCACCACATCGCT,” VP16-R “GAGGATTTGTTTTCGGCGTT,” GAPDH-F “GAAGGTGAAGGTCGGAGTC,” GAPDH-R “GAAGATGGTGATGGGATTTC.”

### Viral infection

Cells at 80%–90% confluency were inoculated with viruses diluted in DMEM at a desired MOI at 37°C for 2 h, washed with PBS and cultured in 1% FBS DMEM.

### Statistical analysis

Data are presented as the mean ± sd, calculated by GraphPad Prism 6.0 software. Two-tailed unpaired Student’s *t*-test was used to calculate *p*-values. *p*-values > 0.05 were marked as “ns,” *p*-values < 0.05 were marked as “*,” *p*-values < 0.01 were marked as “**,” *p*-values < 0.001 were marked as “***,” and *p*-values < 0.0001 were marked as “****.”

## Results

### Oct-1 but not HCF-1 was actively secreted during HSV-1 infection

**Figure 1:**
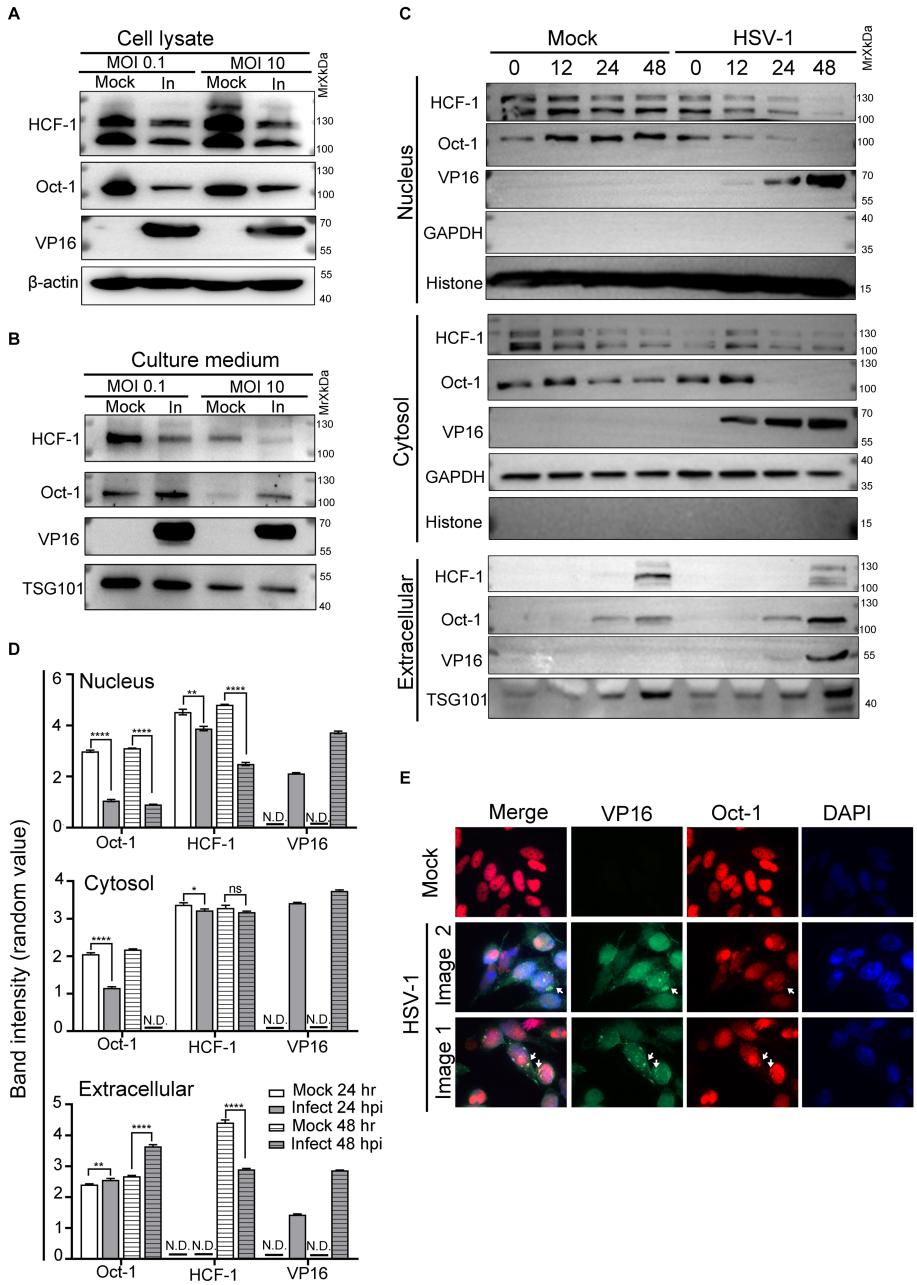
Oct-1 was secreted into the extracellular space during HSV-1 infection. **(A,B)** HEp-2 cells were mock infected or infected with HSV-1 at an MOI of 0.1 for 50 h or an MOI of 10 for 10 h. **(A)** At the indicated times, cell lysates were collected and immunoblotted with anti-HCF-1, anti-Oct-1, and anti-VP16 antibodies, and β-actin served as a control. **(B)** Culture medium was collected, and then cell debris was removed as described in Materials and methods. Proteins secreted into the extracellular space were concentrated by ultracentrifugation and examined by immunoblotting with polyclonal anti-HCF-1, anti-Oct-1, anti-VP16 and anti-TSG101 antibodies. **(C)** HEp-2 cells were mock infected or infected with HSV-1 at an MOI of 0.1. Subcellular fractions and culture medium were collected at the indicated time points and in the panel, 40 μL out of 600 μL cytosolic lysate and 40 μL out of 200 μL nuclear lysate were loaded and immunoblotted with anti-HCF-1, anti-Oct-1 and anti-VP16 antibodies. Histone, GAPDH and TSG101 served as markers for the origin and purity of the samples. **(D)** Band density of Oct-1, HCF-1 and VP16 in different sub-and extra-cellular compartments in panel C was quantified by ImageJ three times independently and plotted as above. **(E)** HEp-2 cells were mock infected or infected with HSV-1 at an MOI of 10. At 12 h post infection, cells were fixed, permeabilized, and reacted with anti-Oct-1 and anti-VP16 antibodies labeled with fluorophores, as indicated. Representative images are shown.

A prior study indicated that successful formation of VIC was critical for initiating transcription of HSV-1 immediate early (IE) genes (Nogueira et al., 2004). As VP16 was carried in the mature virions to facilitate virus amplification, we inquired about the possibility of the other two host components of VIC being actively delivered among cells between rounds of HSV-1 infections. To address this, HEp-2 cells were infected with HSV-1 at an MOI of 0.1 or 10, and the protein levels of Oct-1, HCF-1 and VP16 in the whole cell lysates (Figure 1A) and the extracellular space (Figure 1B) were quantified by immunoblot. While HSV-1 led to reduced cell-associated protein levels of both HCF-1 and Oct-1 after HSV-1 infection, it significantly promoted extracellular secretion of Oct-1 but not HCF-1 at both low and high MOIs (Figure 1B). Since Oct-1 is primarily a nuclear localized transcription factor, we traced the protein levels of Oct-1 in the nucleus, cytosol, and extracellular spaces to examine the temporal and spatial changes in the protein during HSV-1 infection. HEp-2 cells were infected with HSV-1 at an MOI of 0.1, and the subcellular and extracellular distributions of the VIC components were examined by immunoblotting. As shown in Figure 1C and DHSV-1 infection resulted in a gradual reduction in the intracellular levels of both HCF-1 and Oct-1, likely due to disruption of host gene expression by the virus (Shu et al., 2013; Rutkowski et al., 2015; Wang et al., 2020). Interestingly, while both Oct-1 and HCF-1 were secreted by the uninfected HEp-2 cells, extracellular exportation of Oct-1, but not HCF-1, was prominently promoted by HSV-1 infection, starting at 24 hpi and more significantly at 48 hpi (Figures 1C,D). Therefore, we concluded that HSV-1 infection enhanced the extracellular secretion of the VIC component Oct-1 but not HCF-1.

Since VP16 and Oct-1 appeared in the culture medium of HSV-1-infected HEp-2 cells at similar time points post infection, we next examined the subcellular distribution of Oct-1 during HSV-1 infection through immunofluorescence staining. As HSV-1 infection in HEp-2 cells at an MOI of 10 progressed to 10 hpi, costaining of Oct-1 and VP16 was frequently detected in the perinuclear areas, as shown in Figure 1E (white arrows). Note that not all cytosolic Oct-1 and VP16 staining colocalized, and further analysis of the biological significance and consequence of this colocalization is needed.

### HSV-1 from cells bereft of Oct-1 was less efficient in infection

**Figure 2:**
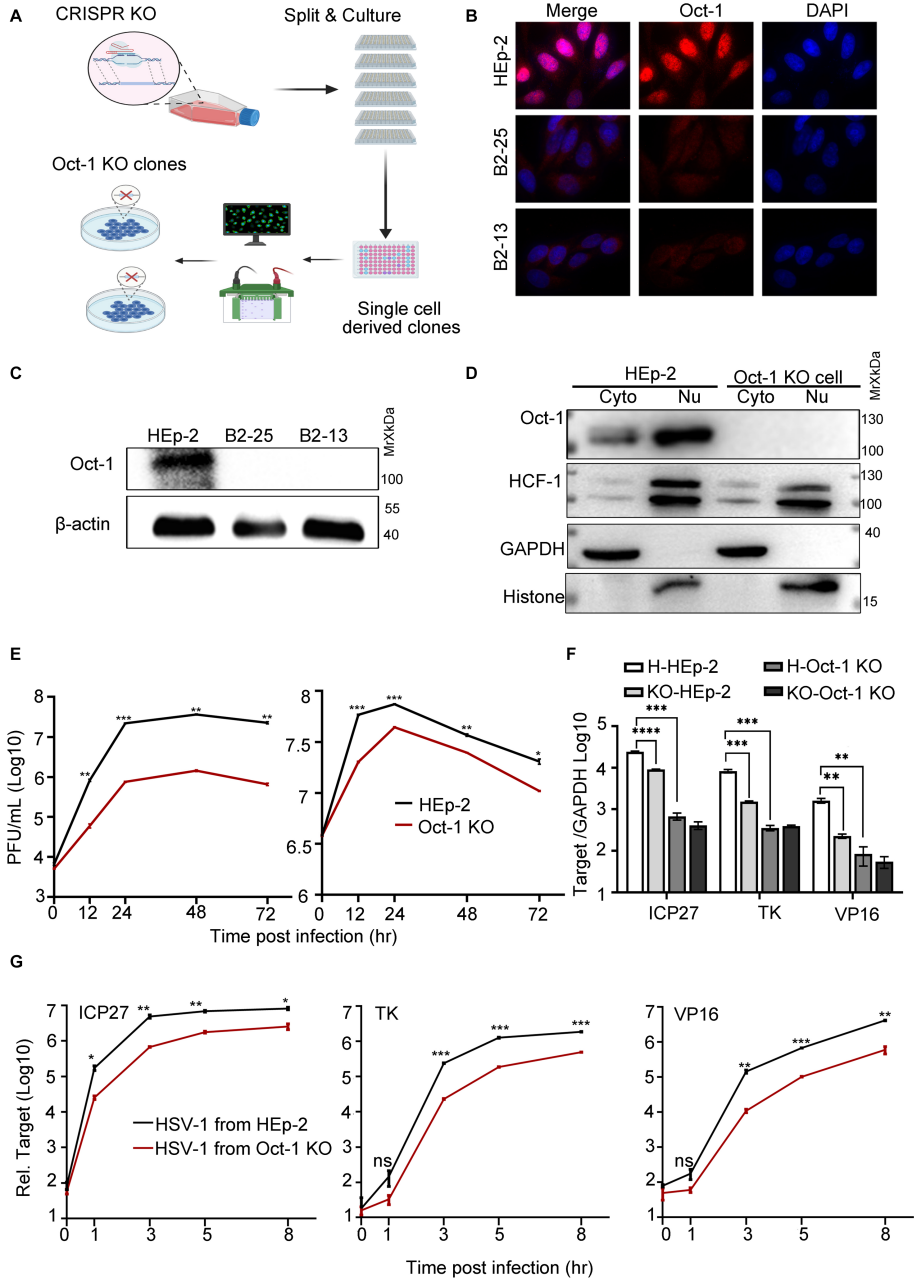
The effect of Oct-1 protein on HSV-1 replication. **(A)** Schematic illustration of the experimental workflow for the generation of single-cell-derived KO clones is described in Materials and methods. **(B)** HEp-2 and two single-cell-derived KO clones (B2-13 and B2-25) were fixed, permeabilized, and reacted with anti-Oct-1 antibody labeled with fluorophores. **(C)** Lysates of parental HEp-2, B2-13 and B2-25 were immunoblotted with anti-Oct-1 antibody. **(D)** Subcellular fractions of HEp-2 and Oct-1 KO cells were collected and 40 μL out of 600 μL cytosolic lysate and 40 μL out of 200 μL nuclear lysate were loaded and immunoblotted with anti-HCF-1 and anti-Oct-1 antibodies. Histone and GAPDH served as markers for the origin and purity of the samples. **(E)** Multicycle growth kinetics of HSV-1 in HEp-2 and Oct-1 KO cells at an MOI of 0.01 (left) or an MOI of 5 (right) by plaque titration of the cell-associated viruses. **(F)** HEp-2 or Oct-1 KO cells were infected with HSV-1 from HEp-2 and Oct-1 KO cells at an MOI of 0.1. KO-and H-represented cell line Oct-1 KO and HEp-2 used for HSV-1 amplification respectively, and HEp-2 and Oct-1 KO indicated the cell lines that were infected with the viruses. **(G)** HEp-2 cells were infected with HSV-1 from supernatant of the indicated infected cell lines. The virus stocks from supernatants were prepared by collecting culture medium of HSV-1 infected cells and centrifuging at 800 g for 5 min. Expression levels of the representative α (ICP27), β (ICP8), and γ (VP16) genes at the indicated time points in panel F and G were quantified by quantitative PCR (qPCR; the Ct values of GAPDH of different experimental groups under the above infection conditions remained steady). *p*-values < 0.05 were marked as “*,” *p*-values < 0.01 were marked as “**,” *p*-values < 0.001 were marked as “***,” and *p*-values < 0.0001 were marked as “****.”

By using CRISPR/Cas9 technology, we developed single-cell-derived monoclonal cell lines deficient in Oct-1 protein derived from HEp-2 cells (Oct-1 KO) to evaluate the role of Oct-1 in both intracellular and intercellular propagation of HSV-1 in human cells (Figure 2A). Complete depletion of Oct-1 protein in the two established Oct-1 KO cell lines (B2-13 and B2-25) was confirmed by immunofluorescence staining (Figure 2B) and immunoblotting (Figure 2C). The monoclonal cell line B2-25 was then used in the rest of this investigation and designated Oct-1 KO. As shown in Figure 2D, there was no detectable Oct-1 protein in each subcellular compartment of Oct-1 KO cells, and the intracellular distribution of HCF-1 and its protein level were not affected in the absence of Oct-1. Consistent with the study of HSV-1 in MEF cells (Nogueira et al., 2004), the virus production of HSV-1 in Oct-1 KO human cells was approximately 20-fold lower than that in wild-type HEp-2 cells at a low MOI, and the growth difference was lessened when the MOI was raised to 5 (Figure 2E), arguing for a nonessential role of Oct-1 and VIC in initiating HSV-1 α gene expression in human epithelial cells, especially at high MOI.

Earlier in this study, we reported that HSV-1 specifically promoted the secretion of Oct-1. We wondered if the virus released from Oct-1 KO cells was less efficient at initiating transcription. As HSV-1 is transmitted both through infectious virus secretions and direct contact with herpes lesions (Goldwich et al., 2011), HSV-1 virus crudes were prepared from either the whole cell lysates (Figure 2F) or the culture medium of the infected HEp-2 or Oct-1 KO cells (Figure 2G), and HEp-2 cells were infected with differentially prepared HSV-1 viruses at an MOI of 0.1. Representative α, β, and γ genes of HSV-1 were quantified by qRT-PCR. While the transcription of representative HSV-1 viral genes of three classes was significantly lower in Oct-1 KO cells than in HEp-2 cells due to the lack of functional Oct-1 and VIC (Figure 2F), it is worth noting that the viruses from Oct-1 KO cells, cell-associated or secreted, transcribed their genomes less effectively than viruses from HEp-2 cells during the next round of infection in HEp-2 cells (Figures 2F,G). In both cases, HSV-1 viruses prepared from cell lysates or secreted in the medium of the infected Oct-1 KO cells were less efficient at activating viral gene transcription in the next infection round under a similar MOI, suggesting the absence of substantial pro-viral contents in these virus preparations from the infected Oct-1 KO cells.

### Oct-1 was packaged into EVs from HSV-1-infected cells

**Figure 3:**
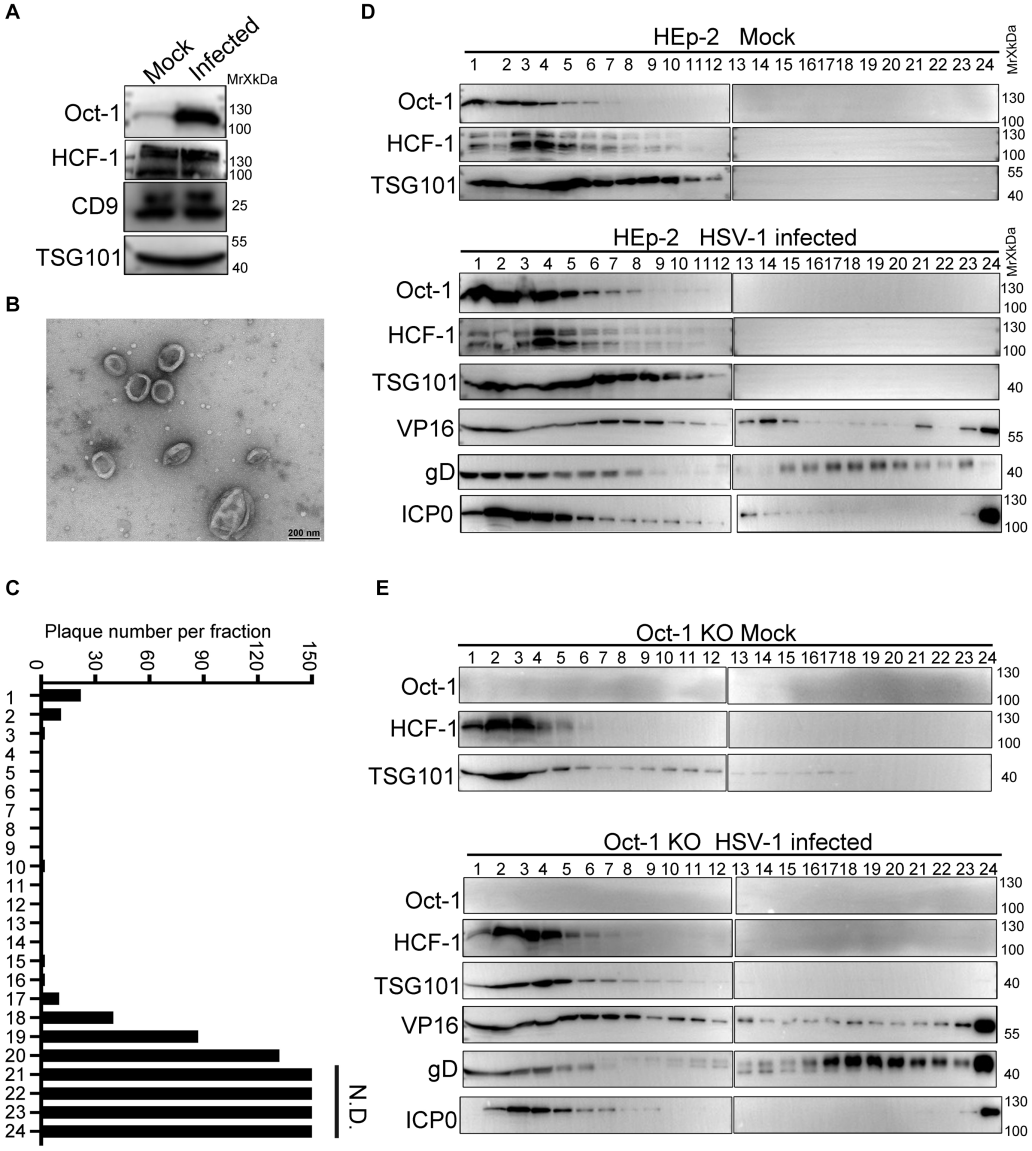
Oct-1 was increasingly packaged into EVs during HSV-1 infection. **(A)** HEp-2 cells were mock infected or infected with HSV-1 at an MOI of 0.1. At 50 hpi, the culture medium was collected, and cell debris was removed as described in Materials and methods. Proteins secreted into the extracellular space were concentrated by ultracentrifugation and examined by immunoblotting with anti-Oct-1 and anti-HCF-1 antibodies. CD9 and TSG101 served as controls. **(B)** EVs were derived from HSV-1(F)-infected HEp-2 cells following procedures described in Materials and methods and analyzed by a JEM-1400 flash electron microscope (JEOL Ltd., Japan). **(C)** Infectious HSV-1 virions in the 24 fractions from panel C were titrated by a plaque assay on Vero cells. The cells exposed to the bottom 2 fractions of the gradient (fractions 23 and 24) were fully infected, so plaques could not be determined. N.D., not determined. **(D,E)** HEp-2 and Oct-1 KO cells were mock infected or infected with HSV-1 at an MOI of 0.1 for 50 h. Culture medium was collected and processed as described in Materials and methods. Extracellular vesicles and HSV-1 virions were separated by iodixanol-sucrose gradient-based ultracentrifugation. Twenty-four fractions, each in 500 μL, were collected from top to bottom from the density gradient, labeled 1 to 24. The proteins present in each fraction were analyzed by immunoblotting with the indicated antibodies.

Various viral and cellular factors are incorporated into the tegument of HSV-1 virions or into extracellular vesicles to facilitate infection (Roizman, 2001; Musarrat et al., 2021). Given that the size of HSV-1 virions is similar to the size of exosomes, which constitute an important part of the extracellular double-lipid wrapped particles, we investigated whether Oct-1 was packed in the infectious virions or in the noninfectious extracellular microvesicles. We utilized an iodixanol/sucrose gradient-based ultracentrifugation method to separate all extracellular vesicles, including HSV-1 virions, into 24 portions numbered in sequence from top to bottom on the iodixanol/sucrose gradient after 16 h of ultracentrifugation (Deschamps and Kalamvoki, 2018; Ma et al., 2022). During the sequential separation of enveloped vesicles from HSV-1 viral particles, we collected the first 1–6 portions, concentrated the particles, and then sent them for analysis by Transmmision electron microscopy (TEM). The TEM results showed that the top solutions contained membrane-wrapped vesicles with diameters ranging from 100 nm to 500 nm, matching the size of exosomes (Figures 3A,B). Titration of the infectious viral particles in the 24 portions confirmed successful separation of EVs (top lanes 1–6) from infectious HSV-1 virions (bottom lanes; Figure 3C).

To further characterize the secretion patterns of Oct-1, HCF-1 and viral protein VP16, HEp-2 and Oct-1 KO cells were mock infected or infected with HSV-1 (0.1 PFU/cell), and the culture medium was collected at 50 hpi and processed as described above. Analysis of the protein contents by immunoblotting with antibodies against Oct-1, HCF-1, TSG101, VP16, gD and ICP0 revealed that while the viral tegument protein VP16 was detected in abundance in both virion-enriched portions and noninfectious extracellular vesicles, Oct-1 and HCF-1 cosedimented only with EVs that were positive for TSG101 in the first 6 lanes but not with the bottom 23 and 24 portions containing HSV-1 virions (Figure 3D). Consistent with Figure 3A, the EV-association level of HCF-1 was not detectably different between the mock-and HSV-1-infected groups. Intriguingly, although all three VIC components were found in the non-virion-containing EVs, depletion of Oct-1 proteins from the cells did not affect the secretion of HCF-1 or VP16 into the EVs, implying that HCF-1 was secreted independently from Oct-1 (Figures 3D,E).

These results indicate that the endogenous Oct-1 protein is increasingly secreted into the extracellular space during HSV-1 infection, and it is associated with non-virion-containing EVs that exhibit exosome characteristics in size, biological membrane markers, and sedimentation velocity in iodixanol-sucrose gradients during ultracentrifugation.

### Oct-1 in EVs was imported into the nucleus of recipient cells and facilitated HSV-1 infection

**Figure 4:**
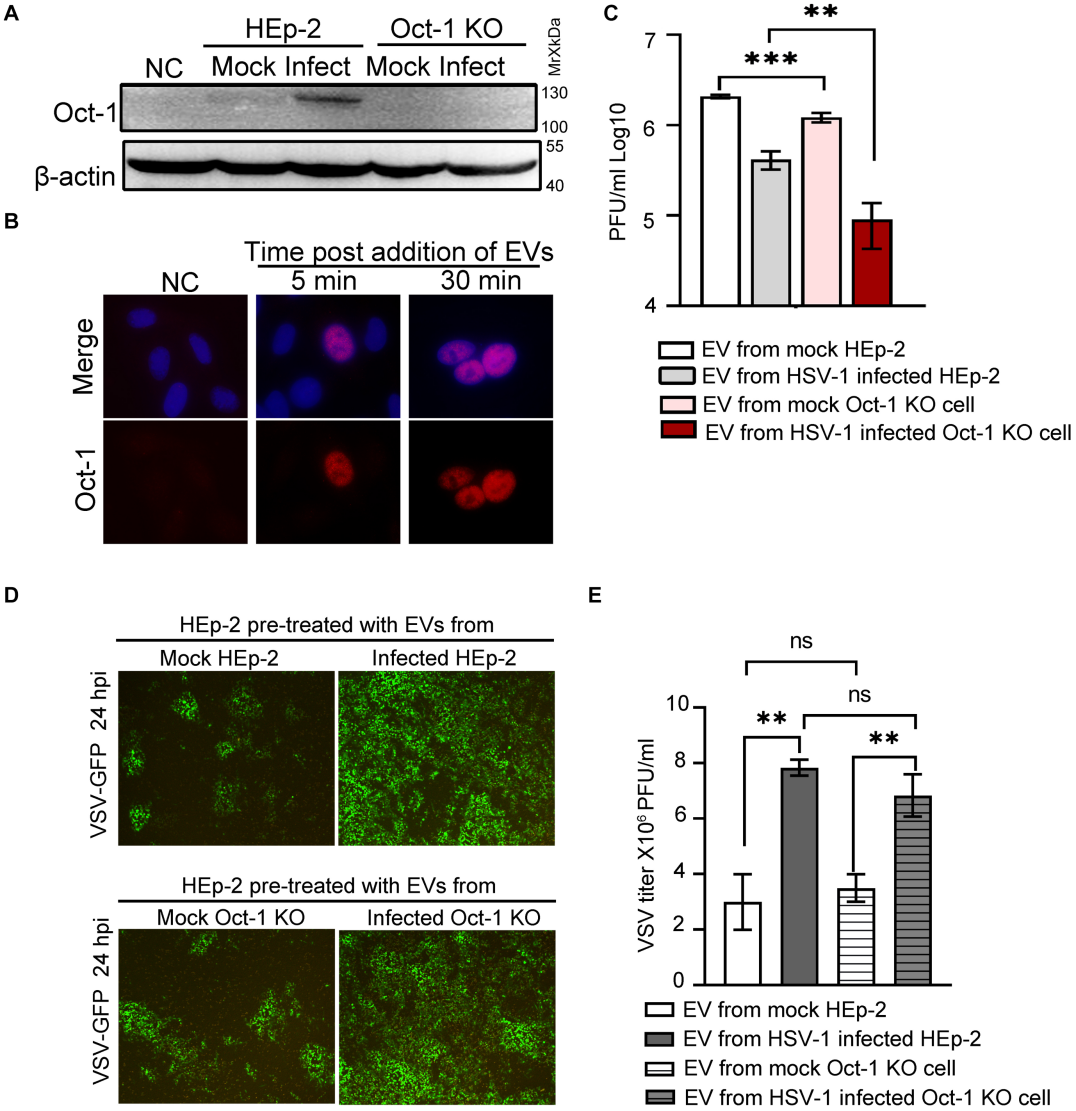
Effect of EVs from HEp-2 and Oct-1 KO cells on HSV-1 or VSV-G replication in recipient HEp-2 cells. **(A–E)** HEp-2 and Oct-1 KO cells were mock infected or infected with HSV-1(F) at an MOI of 0.1 for 50 h, then supernatant was collected and EVs were isolated through iodixanol-sucrose gradient-based ultracentrifugation. The recipient cells were incubated with an equal volume (500 μL) of exosome-enriched fractions (fractions 1 to 6 in Figures 3D–E) for the indicated time. **(A)** The Oct-1 KO cells were incubated with EVs for 2 h, and then the Oct-1 protein level was examined by immunoblotting with anti-Oct-1 antibody. NC represents no exosome treatment. **(B)** A total of 2 × 10^5^ Oct-1 KO cells were incubated with exosome-enriched fractions for 5 min and 30 min, and then reacted with anti-Oct-1 antibody labeled with fluorophores. **(C–E)** A total of 4 × 10^5^ HEp-2 cells incubated with EVs were infected with 4,000 PFU of HSV-1 or 40,000 VSV-GFP at 37°C for 2 h. **(C)** At 24 hpi, cell-associated HSV-1 were titrated by a plaque assay. **(D,E)** At 24 hpi, green fluorescent protein (GFP) levels in HEp-2 cells treated with EVs were photographed under a fluorescence microscope **(D)**, and VSV-GFP viruses from the supernatant were titrated by a plaque assay **(E)**. *p*-values < 0.01 were marked as “**,” *p*-values < 0.001 were marked as “***”.

Next, we examined whether EV-associated Oct-1 protein could be internalized by recipient cells. We first separated HSV-1 virions from EVs by the iodixanol/sucrose gradient ultracentrifugation method and collected the top 6 portions, designated EVs for the studies in this section. When Oct-1 KO cells were incubated with equal volumes of EVs from mock-or HSV-1-infected HEp-2 or Oct-1 KO cells, significantly more Oct-1 protein was detected in the cells incubated with the EVs from HSV-1-infected HEp-2 cells than in those that received EVs from the mock-infected HEp-2 group or from either Oct-1 KO group (Figure 4A). Importantly, Oct-1 delivered by EVs from HSV-1-infected HEp-2 cells was detected in the nucleus of recipient cells (Oct-1 KO) as early as 5 min post EV incubation, implying high compatibility between the secretions from infected cells and naïve cells and normal bioactivity of EV-associated Oct-1 (Figure 4B).

To investigate the virological consequence of depletion of Oct-1 protein and its potentially copacked factors in EVs from HSV-1-infected cells, HEp-2 cells were first incubated with EVs from mock-and HSV-1-infected HEp-2 and Oct-1 KO cells and exposed to HSV-1 at an MOI of 0.01. Cell-associated HSV-1 was collected at 24 hpi and titrated by a plaque assay. Consistent with our previous report and reports by others (Deschamps and Kalamvoki, 2018; Ma et al., 2022) EVs from HSV-1-infected cells exerted an inhibitory effect against the next round of HSV-1 infection in general compared with EVs from mock-infected cells for both cell lines (Figure 4C). In particular, EVs from Oct-1 KO cells, infected with HSV-1 or not, were significantly less supportive of subsequent HSV-1 infection than their matching samples from HEp-2 cells, emphasizing the value of noninfectious EV-mediated intracellular delivery of Oct-1 for HSV-1.

Since the secretion of the other essential VIC component HCF-1 remained unaffected during HSV-1 infection, we wondered whether the enriched Oct-1 packaging in EVs has general pro-viral activity. HEp-2 cells were pretreated with EVs from four experimental groups as described above and infected with a recombinant vesicular stomatitis virus carrying a GFP reporter gene (VSV-GFP). At 24 hpi, cells were imaged, and VSV virus in the culture medium was titrated (Figures 4D,E). Replication of VSV in HEp-2 cells preincubated with two groups of HEp-2-derived EVs was indistinguishable from that in Oct-1 KO cells in GFP expression and virus titer, strengthening the idea that Oct-1 plays a specific pro-viral role in HSV-1. Surprisingly, both EVs from HSV-1-infected HEp-2 cells and Oct-1 KO cells significantly promoted VSV-GFP infection (Figures 4D,E), suggesting the possibility for future research to determine whether EVs from HSV-1-infected cells promote infection by certain viruses.

## Discussion

In this report, we established a single-cell-derived Oct-1 KO cell line on the background of HEp-2 cells. Based on this, we found that both cell-associated virus and secreted virus from HSV-1-infected Oct-1 KO cells were significantly less efficient in initiating the next round of infection in wild type (wt) HEp-2 cells, implying that the presence of Oct-1 in prior infection is critical for the two primary modes of HSV-1 transmission, cell-free release (CFR) and cell–cell spread (CCS; Rice, 2021). Further investigation showed that HSV-1 selectively promoted the secretion of endogenous Oct-1 during infection in noninfectious virion-containing EVs but not HCF-1, the other key component of the VIC complex. Intercellular communication between EVs allows Oct-1 in EVs to be efficiently internalized by uninfected cells, imported into the nucleus, and promote HSV-1 infection. In this report, we identified an Oct-1-mediated pro-HSV-1 pathway through EV-mediated intercellular communication, and our findings lead to discussions in the following directions.

### HSV-1 infection selectively promoted EV-mediated secretion of the nuclear protein Oct-1

Viruses promote both viral and host factor secretion through EVs, frequently in a cell-type-and infection stage-dependent manner, implying intentional manipulation of the process by the two players (Schorey et al., 2015). Both pro-viral and antiviral host factors have been found in these extracellular cargo carriers. For example, HIV is known to alter the composition and function of EVs, leading to the secretion of infection-promoting host proteins such as virus receptors CCR5 and CXCR4, metalloprotease ADAM17 and other proinflammatory factors, and host restrictive factors such as APOBEC3G and TRIM5α (Mack et al., 2000; Rozmyslowicz et al., 2003; Khatua et al., 2009; Lee et al., 2016; Ciccosanti et al., 2021). EVs released by Kaposi’s sarcoma-associated herpesvirus (KSHV/HHV-8) are enriched in metabolic proteins and in proteins affecting the immune system to facilitate their persistence (Jeon et al., 2019). In the case of HSV-1, studies have shown that the virus enhances EV-dependent communication of various viral factors and have identified several host restrictive proteins enriched in these double-lipid structures, including STING, CD63 and Sp100A (Deschamps and Kalamvoki, 2018; Dogrammatzis et al., 2019; Ma et al., 2022). In this investigation, we identified Oct-1 as a pro-viral host protein that was increasingly wrapped in EVs from HSV-1-infected cells.

Interestingly, Oct-1 is a transcription factor that is mainly localized in the nucleus (Figures 1C, 2D). Prior studies have shown that post-translational modifications, specific protein binding and certain cellular stress responses could modulate the nuclear-cytosolic shuttling of Oct-1 and that HSV-1 infection modifies Oct-1 late in lytic infection through an unknown mechanism, leading to its reduced DNA-binding affinity (Advani et al., 2003; Malhas et al., 2009; Boubriak et al., 2017). Although our data indicated that Oct-1 and VP16 colocalized at the perinuclear area at later stages of HSV-1 infection (Figure 1E), it remains an enigma how HSV-1 promotes the transportation of this nuclear protein through cellular secretion pathways and/or whether the process takes advantage of the gradual rupture of the nuclear envelope induced by HSV-1 infection (Arii et al., 2018; Arii, 2021). The finding was nonetheless not entirely surprising given the recent reports of enhanced EV packaging of nuclear ribonucleoproteins (hnRNPs), histones, and transcription factors during retrovirus infection (Nair et al., 2018; Barclay et al., 2019; Zietzer et al., 2020).

### Potential impacts of Oct-1 secretion beyond its role in subsequent HSV-1 lytic replication

In the following discussion, we discuss whether enhanced EV incorporation of Oct-1 has additional functions other than facilitating alpha gene transcription of HSV-1. Clearly, HSV-1 infection promoted the secretion of Oct-1 but not HCF-1 (Figures 1B,C, 3A,D), and the HCF-1 level in the EVs was affected by neither HSV-1 infection nor the knockout of Oct-1 (Figures 3A,D,E). This is a rather interesting phenomenon, as VP16, a tight HCF-1-interacting viral protein that was detected in abundance in these EVs, did not enhance EV-wrapping of HCF-1 during infection (Figures 3D,E). Prior studies have shown that HCF-1 is an essential factor for IE gene expression and that depletion of HCF-1, but not Oct-1, results in abrogation of IE gene expression and suggests that the cytosolic-nuclear shuttling of HCF-1 in neurons contributes to the establishment of and reactivation from latency by HSV-1 (Arbuckle et al., 2023). In conclusion, it appears that the virus induces EV-packaging of Oct-1 independent of HCF-1, and further investigation is necessary to determine whether EV-associated Oct-1 performs any other pro-viral functions besides transcription initiation of lytic genes.

### Complex compositions of HSV-1-induced EVs with opposing activities

HSV-1 infection induces the secretion of heterogeneous EVs that are distinct in size, origin, and composition (Kalamvoki and Deschamps, 2016). The major cellular pathways involved in the biogenesis of EVs include ESCRT-dependent and ESCRT-independent secretion of intraluminal vesicles (ILVs) within endosomal multivesicular bodies (MVBs), plasma membrane budding of microvesicles and plasma budding of apoptotic bodies (Gurung et al., 2021). Previously, Christos et al. showed that during HSV-1 infection, viral components were found in pro-viral and ESCRT-positive EVs, while the antiviral protein STING was enriched in viral inhibitory EVs that were positive for CD63, CD81 and other tetraspanins (Dogrammatzis et al., 2021). Examples of the EV-associated pro-infection viral factors include ICP0 that targets multiple signaling molecules in RLR/TLR, DNA sensing, JAK-STAT, DNA damage response (DDR), ER stress, autophagy and apoptosis signaling pathway (Melroe et al., 2004; Halford et al., 2006; Melroe et al., 2007; van Lint et al., 2010; Orzalli et al., 2012; Johnson et al., 2013; Zhang et al., 2013; Zhu and Zheng, 2020), VP16 that abolishes IFN-β production by inhibiting NF-κB activation and blocking complex formation of IRF3-CREB (Xing et al., 2013), and Us11 that downmodulates RLR signaling pathways via targeting MDA5 and RIG-I (Xing et al., 2012), degrades TBK1 (Liu et al., 2018) and suppresses ISGs expression (Poppers et al., 2000; Sànchez and Mohr, 2007; Liu et al., 2019). And EV-associated antiviral factors identified to date include STING and Sp100A (Malhas et al., 2009; Deschamps and Kalamvoki, 2018; Ma et al., 2022). In this report, total EVs released from HSV-1-infected cells promoted VSV replication in an Oct-1-independent manner, implying that these extracellular vesicles may be involved in the synergy of virus coinfection, at least in the case of VSV (Figures 4D,E). Interestingly, Christos et al. observed that treatment of respiratory syncytial virus (RSV)-infected cells (at 48 hpi) with CD63+ EVs stimulated by HSV-1 infection mildly reduced intracellular RSV genome copies (<2-fold; Dogrammatzis et al., 2021). The seemingly contradictory phenotypes could be explained by the fact that the prior work used the strong anti-viral population of HSV-1-stimulated EVs (CD63+ STING+ EV portion), and our study assessed the effects of total HSV-1-induced EVs on VSV. Moreover, two sets of infection experiments were designed differently to serve distinct investigation purposes. In this report, cells were treated with HSV-1-induced EVs before being infected with VSV to evaluate the impact of HSV-1 infection-promoted EVs on a secondary infection, while Christos et al. sought to determine if CD63+ EVs from HSV-1-infected cells could be utilized as a broad antiviral strategy/treatment.

Nevertheless, these observations call for further investigate if it is a general principle that HSV-1 takes advantage of cellular cargo sorting pathways to differentially allocate pro-viral and anti-viral factors into distinct EVs. An extension and related inquiry to this hypothesis is whether the virus modulates its cell tropism within the infected tissue by delivering distinct contents into different cell types, as these EVs are decorated with diverse sets of surface markers that attach/receive efficiently to different recipient cells (Ohno et al., 2013; French et al., 2017; Kamerkar et al., 2017).

## Conclusion

EVs play an active role in intercellular communication. HSV-1 has been reported to drastically promote the secretion of these double-lipid vesicles during infection. Host proteins, such as STING, CD63, and Sp100 have been reported to be loaded in EVs and increasingly secreted during HSV-1 infection. However, they all mediate antiviral effects against HSV-1 and viruses in general. No pro-viral host factor has been hitherto identified. It would be surprising if HSV-1 did not smuggle host proteins to its own advantage during this process. The present study shows that the host transcription factor Oct-1 is actively exported into EVs during HSV-1 infection and facilitates the initiation of viral transcription. This investigation reports one of the first pro-viral host proteins packed into EVs during HSV-1 infection and underlines the heterogenetic nature and complexity of these noninfectious double-lipid particles.

## Data availability statement

The raw data supporting the conclusions of this article will be made available by the authors, without undue reservation.

## Author contributions

PX designed the project and wrote the manuscript. YM and XD participated and performed most of the experiments and helped to revise the manuscript. LZ helped with EVs purification, subcellular fractionation, and manuscript preparation. HD performed some of the immunofluorescence staining experiments. All authors contributed to the article and approved the submitted version.

## Funding

This project was supported by the National Key Research and Development Program of China (2022YFC2305400), the National Natural Science Foundation of China (no. 31870157), the Shenzhen Science and Technology Innovation Program (JCYJ20180307151536743 and KQTD20180411143323605), and Natural Science Foundation of Shenzhen City(JCYJ2022050145810023).

## Conflict of interest

The authors declare that the research was conducted in the absence of any commercial or financial relationships that could be construed as a potential conflict of interest.

## Publisher’s note

All claims expressed in this article are solely those of the authors and do not necessarily represent those of their affiliated organizations, or those of the publisher, the editors and the reviewers. Any product that may be evaluated in this article, or claim that may be made by its manufacturer, is not guaranteed or endorsed by the publisher.
